# Predicting Protein–Protein Interactions Based on Ensemble Learning-Based Model from Protein Sequence

**DOI:** 10.3390/biology11070995

**Published:** 2022-06-30

**Authors:** Xinke Zhan, Mang Xiao, Zhuhong You, Chenggang Yan, Jianxin Guo, Liping Wang, Yaoqi Sun, Bingwan Shang

**Affiliations:** 1 School of Information Engineering, Xijing University, Xi’an 710123, China; przhanxinke@gmail.com (X.Z.); guojianxin@xijing.edu.cn (J.G.); wangliping@xijing.edu.cn (L.W.); bingwanshang@163.com (B.S.); 2 Sir Run Run Shaw Hospital, Zhejiang University, Hangzhou 310016, China; 3 School of Computer Science, Northwestern Polytechnical University, Xi’an 710129, China; 4 School of Automation, Hangzhou Dianzi University, Hangzhou 310018, China; cgyan@hdu.edu.cn (C.Y.); syq@hdu.edu.cn (Y.S.); 5 School of Mechanical, Electrical and Information Engineering, Shandong University, Weihai 264209, China

**Keywords:** locality preserving projections, rotation forest, PSSM, SVM, KNN

## Abstract

**Simple Summary:**

Due to most traditional high-throughput experiments are tedious and laborious in identifying potential protein–protein interaction. To better improve accuracy prediction in protein–protein interactions. We proposed a novel computational method that can identify unknown protein–protein interaction efficiently and hope this method can provide a helpful idea and tool for proteomics research.

**Abstract:**

Protein–protein interactions (PPIs) play an essential role in many biological cellular functions. However, it is still tedious and time-consuming to identify protein–protein interactions through traditional experimental methods. For this reason, it is imperative and necessary to develop a computational method for predicting PPIs efficiently. This paper explores a novel computational method for detecting PPIs from protein sequence, the approach which mainly adopts the feature extraction method: Locality Preserving Projections (LPP) and classifier: Rotation Forest (RF). Specifically, we first employ the Position Specific Scoring Matrix (PSSM), which can remain evolutionary information of biological for representing protein sequence efficiently. Then, the LPP descriptor is applied to extract feature vectors from PSSM. The feature vectors are fed into the RF to obtain the final results. The proposed method is applied to two datasets: *Yeast* and *H. pylori*, and obtained an average accuracy of 92.81% and 92.56%, respectively. We also compare it with *K* nearest neighbors (KNN) and support vector machine (SVM) to better evaluate the performance of the proposed method. In summary, all experimental results indicate that the proposed approach is stable and robust for predicting PPIs and promising to be a useful tool for proteomics research.

## 1. Introduction

Protein–protein interactions (PPIs) play a crucial role in almost all cellular processes and functions, such as DNA transcription and replication, immune response, signal transduction, and gene expression [1,2]. Thus, detecting and characterizing potential protein interactions correctly are significant for understanding the properties of biological processes. Recently, a number of innovative high-throughput biological experimental technologies, including yeast two-hybrid screen (Y2H) [3,4], protein chip [5], and tandem affinity purification tagging (TAP) [6], and other methods have been proposed to detect the interaction between proteins systematically. With the development of biotechnology, the number of PPI data is quickly accumulating. For this reason, multiple databases have been built to record PPI data efficiently. Biomolecular Interaction Network Database (BIND) [7], Database of Interacting Proteins (DIP) [8], and the Molecular Interaction database (MINT) [9] are mainly used databases by researchers. However, there still exist some drawbacks to traditional high-throughput methods, such as being costly and labor-intensive and it will raise a high rate of false positives. The known PPI pairs which have been validated through biological experiment methods only account for a small portion of the whole PPI network [10,11]. As a result, developing a novel computational method is conducive to inferring potential PPIs.

Up to now, a great number of computational techniques have been proposed for predicting potential PPIs [12,13,14]. Generally, some existing methods for predicting PPIs typically can be treated as binary classification problems which adopt different features to represent protein pairs [15,16,17]. Different feature sources or protein attributes like protein domains, phylogenetic profiles, and protein structure information are employed to detect potential protein interactions. There are also exist methods that utilize interaction information from several different protein features [18,19]. However, these approaches are not easy to implement unless pre-knowledge of protein pairs can be available.

Recently, a couple of computational methods mainly based on protein sequences have been proposed since protein sequences are the easiest to obtain [20,21,22]. Many researchers have engaged in the development of a sequence-based method for detecting potential PPIs [23,24,25,26], and a variety of experimental results indicated that it is sufficient to predict PPI using the information of amino acid sequences alone [27,28,29,30,31]. For instance, Xia et al. [32] proposed that the Moran autocorrelation descriptor can effectively depict the level of the correlation between two protein sequences of specific physicochemical property and use rotation forest to predict PPIs. Shen et al. [33] proposed a computational method that extracts features by utilizing the conjoint triad (CT) which considered the local environments of residues, and then using a support vector machine (SVM) to predict PPIs; this method achieved the result of average accuracy of 83.9%. You et al. [34] developed a novel computational method that used multi-scale continuous and discontinuous (MCD) to represent protein sequence and achieved an excellent result in the *Yeast* dataset. Chen et al. [35] reported an approach that uses XGBoost to reduce feature noise and adopts StackPPI that several ensemble classifiers to detect the interaction of protein pairs. Zhao et al. [36] proposed an ensemble method and the results of the proposed method obtained good performance. Yousef et al. [37] developed a sequence-based, fast, and adaptive PPIs prediction method, which employed principal component analysis (PCA) as a proper feature extraction method and utilized adaptive learning vector quantization (LVQ) to predict different PPI datasets. This method achieved an average accuracy of 93.88% and 90.03% on *S. cerevisiae* and *H. pylori*, respectively. Wang et al. [38] reported an approach only using the information of protein sequence; the approach combined continuous and discrete wavelet transforms and weight sparse representation-based classifier for predicting PPIs. Zahiri et al. [39] proposed a novel evolutionary-based algorithm called PPIevo, which extracts features from PSSM for predicting protein–protein interactions. In general, previous works illustrate that the feature extraction method and classification are the two most important steps to predicting PPIs.

In this paper, by fully using evolutionary information of protein sequence, we report a novel computational method, which obtaining numerical representation by Position Specific Scoring Matrix (PSSM), extracting feature vector by using locality preserving projections (LPP), and predicting by rotation forest (RF) classifier. More Specifically, the first step is transforming protein sequence into numerical representation, PSSM. Second, each PSSM can be extracted by LPP, and obtained a low-dimensional feature vector. Finally, the feature descriptors are fed into the RF classifier for inferring potential protein–protein interactions. Two datasets, *Yeast* and *H. pylori*, are applied in our proposed method; the five-fold cross-validation results of average accuracy are 92.81% and 92.56%, respectively. The performance of our proposed approach is better than the support vector machine (SVM), *K* nearest neighbors. We also performed extensive experiments on four cross-species independent datasets. Experimental results show that the proposed method outperforms other existing methods and we hope this approach can provide a solution for inferring potential PPIs.

## 2. Materials and Methods

### 2.1. Datasets

The dataset can help us know whether the performance of the proposed method is good or not. In this study, we adopt two benchmark datasets to evaluate the model. Firstly, from the DIP database [8], the *Yeast* PPI dataset was collected. For enhancing credibility, the length of protein pairs that were less than 50 residues and protein pairs that have more than forty percent sequence identity will be directly removed. Thus, the *Yeast* dataset was constructed by 11,888 protein pairs, including a positive dataset of 5594 protein pairs and a negative dataset of 5594 protein pairs. The second dataset—the *H. pylori* PPI dataset—is described by Martin et al. [40]; the whole dataset is constructed by 2916 protein pairs (1458 interacting pairs and 1458 non-interacting pairs).

### 2.2. Position Specific Scoring Matrix

Position-Specific Scoring Matrix (PSSM) [41] is widely used for transform biological sequence into numerical representation [42,43]. Given a protein sequence with length *N*, The PSSM can be represented as follows:

(1) $$D=\left[\begin{array}{cccc} \alpha_{1,1} & \alpha_{1,2} & \cdots & \alpha_{1,20}\\ \alpha_{2,1} & \alpha_{2,2} & \cdots & \alpha_{2,20}\\ \vdots & \vdots & \ddots & \vdots \\ \alpha_{N,1} & \alpha_{N,2} & \cdots & \alpha_{N,20} \end{array}\right]$$

where $\alpha_{i,j}$ means the probability of the *i*th residue being mutated into type *j* of 20 native amino acids during the evolutionary process of the protein from multiple sequence alignments. In this step, employing the tool mentioned in [44] can convert each protein sequence into PSSM. 

### 2.3. Locality Preserving Projections

We aim to extract the feature vectors, which mainly reduce the dimensional of the original matrix to reduce the influence of noise. In this section, Locality Preserving Projections (LPP) algorithm [45] is adopted for extracting feature vectors in each PSSM. It adopts a linear approximation to the Laplace characteristic map for obtaining the structure feature between neighboring. Thus, it is widely used for data processing and analysis application. Given a training sample $X={\left[x_{1},x_{2},\ldots ,x_{n}\right]}^{T}\in R^{D}$, where *D* is the feature dimension, and *n* denotes the feature vectors of each sample. Then for projecting the high-dimensional input dataset *X* into a low-dimensional dataset $Y=\left[y_{1},y_{2},\ldots ,y_{n}\right]$, it is necessary to seek a projection matrix *W*. The objective function of LPP is defined as

(2) $$argmin\overset{m}{\underset{i,j=1}{\sum }}{\left(y_{i}-y_{j}\right)}^{2}P_{ij}$$

where

(3) $$P_{ij}=\{\begin{array}{c} \exp\left(-\frac{{\left\|x_{i}-x_{j}\right\|}^{2}}{t}\right),\;\;\;\;\;x_{i}\;and\;x_{j}\;is\;linked\;\;\;\;\;\\ \;\;\;\;\;\;\;\;\;\;\;\;\;\;\;\;\;\;\;\;\;\;0,\;\;\;\;\;\;\;\;\;\;\;\;\;\;\;\;\;\;\;x_{i}\;and\;x_{j}\;is\;not\;linked\;\;\; \end{array}$$

(4) $$y_{i}=w^{T}x_{i}$$

where $P_{ij}$ is the heat kernel, *w* denotes a transformation vector, and in Equation (3), the parameter *t* means scale size. Thus, we can define the distance formula is shown as follows:

(5) $$d(x_{i},x_{j})=\|x_{i}-x_{j}\|$$

Then, it is necessary to minimize the projection matrix *W*. The steps are defined as:

(6) $$\begin{array}{c} \frac{1}{2}\sum_{i,j}{\left(y_{i}-y_{j}\right)}^{2}P_{ij}=\frac{1}{2}\sum_{i,j}{\left(w^{T}x_{i}-w^{T}x_{j}\right)}^{2}P_{ij}\\ =w^{T}X\left(D-W\right)X^{T}w=w^{T}XLX^{T}w \end{array}$$

where *D* is a diagonal matrix, $Dii=\sum_{j=1}^{n}W_{ij}$ and L=D−W represent the Laplacian matrix. The constraint is:

(7) $$w^{T}XDX^{T}w=1$$

Finding a transfer matrix *w*, the following generalized eigenvalue problem:

(8) $$XLX^{T}w=\lambda XDX^{T}w$$

Solving the question in Equation (8), we can obtain all the eigenvalues and eigenvectors corresponding, *k* eigenvalues: $\lambda_{0},\lambda_{1},\ldots ,\lambda_{k-1}$ are sorted from small to large. $\left\{w_{0},w_{1},\ldots ,w_{k-1}\right\}$ denotes the corresponding characteristic vectors. First, *l* eigenvectors are selected to form the projection matrix $W=[w_{0},w_{1},\ldots ,w_{l-1}]$. As a result, the embedding descriptors are

(9) $$x_{i}\to y_{i}=W^{T}x_{i}$$

### 2.4. Rotation Forest

Rodriguez et al. [46] proposed rotation forest (RF) is a typical ensemble learning algorithm that is widely used in the classification task. In the RF algorithm, it first divides the feature set into *K* subsets by randomly combining the features of the sample. Principal component analysis (PCA) is then used to transform the data and retain the accuracy of the original data. As a result, RF improves classification performance by amplifying the differences between base classifiers.

Let training sample set *S* be an M×m matrix. *m* represents the feature vector length of each training sample. Let *X* be the feature set, and $Y={\left(y_{1},y_{2},\ldots ,y_{n}\right)}^{T}$ are corresponding labels. Assuming *L* decision tree, which can be denoted as $\left[Q_{1},Q_{2},\ldots ,Q_{L}\right]$, respectively. In this algorithm, the complete feature set will be divided into *K* subsets equally and randomly. A single classifier $Q_{i}$ of processing steps can be summarized as follows:

(1) Set *X* is randomly divided into *K* disjoint subsets; each subset contains the number of features is C=M/K.
(2) Form a new matrix $S_{i,j}$ by choosing the corresponding column of the feature in the subset $Q_{i,j}$ from the training dataset *S*. And applying a bootstrap sampling technique from seventy-five percent of the original training dataset *S* to generate a new matrix $S_{j,j}^{\prime }$.
(3) Employ *C* feature by adopting the PCA method in matrix $S_{j,j}^{\prime }$. The principal component coefficients are stored in $T_{i,j}$, which can be represented as $\gamma_{ij}^{\left(1\right)},\ldots ,\gamma_{ij}^{\left(C_{j}\right)}$.
(4) Construct a sparse rotation matrix $F_{i}$, in which matrix $S_{i,j}$ contain coefficients. The matrix $F_{i}$ can be defined as:
  (10) $$F_{i}=\left[\begin{array}{cccc} \gamma_{ij}^{\left(1\right)},\ldots ,\gamma_{ij}^{\left(C_{j}\right)} & 0 & \cdots & 0\\ 0 & \gamma_{ij}^{\left(1\right)},\ldots ,\gamma_{ij}^{\left(C_{j}\right)} & \cdots & 0\\ \vdots & \vdots & \ddots & \vdots \\ 0 & 0 & \cdots & \gamma_{ij}^{\left(1\right)},\ldots ,\gamma_{ij}^{\left(C_{j}\right)} \end{array}\right]$$

Given a test sample *x* in the prediction phase, $d_{i,j}\left(xF_{i}^{a}\right)$ is the probability. The confidence of a class can be computed by the average combined method, and the formula is shown as:

(11) $$\mu_{j}=\frac{1}{L}\sum_{i=1}^{L}d_{i,j}\left(xF_{i}^{a}\right)$$

Thus, the greatest possible can be easily assigned, and the final predicted label can be obtained.

## 3. Results and Discussion

### 3.1. Evaluation Criteria

To more intuitively evaluate the predictive performance of the model, the criteria were evaluated using the classification precision (*Prec.*), accuracy (*Accu.*), Matthews correlation coefficient (*MCC*) and sensitivity (*Sen.*) these are defined respectively by

(12) $$Accu.=\frac{TP+TN}{TP+TN+FP+FN}$$

(13) $$Prec.=\frac{TN}{TN+FP}$$

(14) $$Sen.=\frac{TP}{TP+FN}$$

(15) $$MCC=\frac{TP\times TN-FP\times FN}{\sqrt{\left(TP+FN\right)\times \left(TN+FP\right)\times \left(TN+FN\right)\times \left(TP+FP\right)}}$$

where *FP* is false positive, *TN* is true negative, *FN* is false negative, and *TP* is true positive. The ROC curve is a curve that describes relative trade-offs between *TP* and *FP*. The x-axis of the ROC curve is defined as the false positives rate (*FPR*) or 1-specificity, and the y-axis is defined as the true positives rate (*TPR*) or sensitivity.

(16) $$TPR=\frac{TP}{\left(TP+FN\right)}$$

(17) $$FPR=\frac{FP}{\left(FP+FN\right)}$$

The ROC curve of the best possible contains a point very close to coordinate (0,1) or in the upper left corner of the ROC space, which represents the highest specificity and sensitivity. In our paper, the area under the ROC curve (AUC) is computed, which shows the performance of the proposed method in numerical form.

### 3.2. Prediction Ability Assess

**Figure 1:**
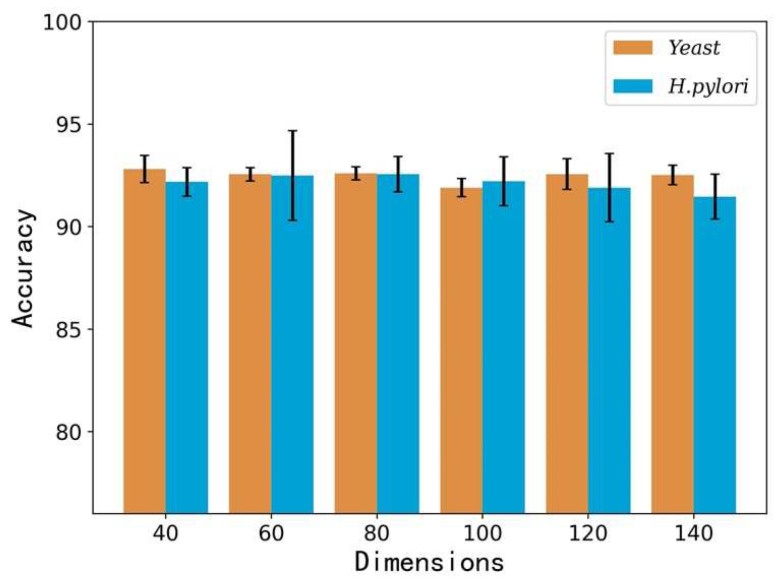
The accuracy performance of the *Yeast* and *H. pylori* datasets.

In this section, we carried out our proposed method on two datasets: *Yeast* and *H. pylori*. Meanwhile, the 5-fold cross-validation method is also adopted to assess the reported method and avoid over-fitting in the experiments. By doing this, five training models would be generated on five groups of training datasets. To obtain the best feature vector representation in the LPP algorithm, we implement a variety of feature vectors with different dimensions (40-dimensional, 60-dimensional, 80-dimensional, 100-dimensional, 120-dimensional, and 140-dimensional) to predict protein interactions to obtain the best feature representation. We repeat this experiment several times and the optimization results can be seen in Table 1. When adopting 40-dimensional feature vectors in the *Yeast* dataset, the result of accuracy achieved 92.81%, and when employing 80-dimensional feature vectors in the *H. pylori* dataset, the result of accuracy yielded 92.56%. We also plotted the accuracy performance of the *Yeast* and *H. pylori* datasets in Figure 1, which clearly illustrate that the best performance can be obtained by using 40-dimensional feature vectors on *Yeast* dataset and 80-dimensional feature vectors on the *H. Pylori* dataset. However, it is worth noting that there has been a decrease from 80-dimensional to 100-dimensional in the *Yeast* dataset. We thought that when we extract feature vectors from PSSM, 100-dimensional feature vectors have more redundant and noise information than 80-dimensional feature vectors. The accuracy gap between 80-dimensional and 100-dimensional is 0.70%. Moreover, the accuracy score is the main criteria we focused on. Thus, 40-dimensional feature vectors are selected on the *Yeast* dataset and 80-dimensional feature vectors are adopted on the *H. pylori* dataset.

In the rotation forest algorithm, there are two main parameters in the rotation forest classifier: *K* and *L*. where *K* represents the number of feature subsets, *L* denotes the number of decision trees. We select the best parameter after the experiment and set *K, L* as 5, 5, respectively. The results of the two datasets were shown in Table 2 and Table 3.

**Figure 2:**
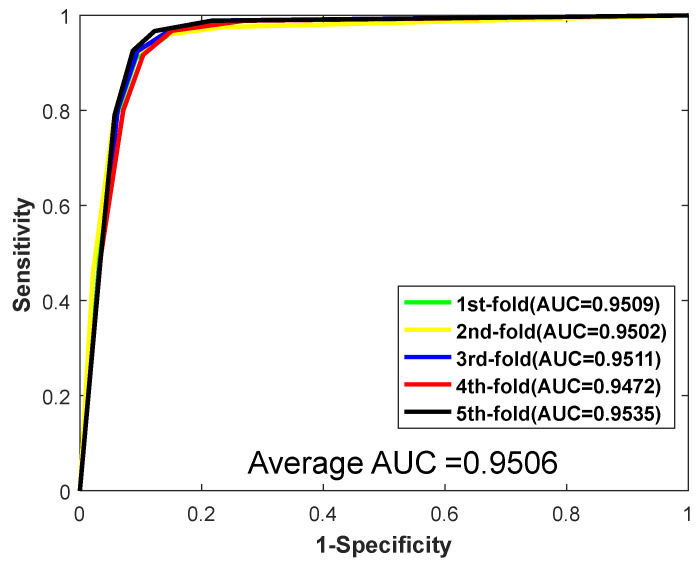
ROC curves yielded by RF on *Yeast*.

**Figure 3:**
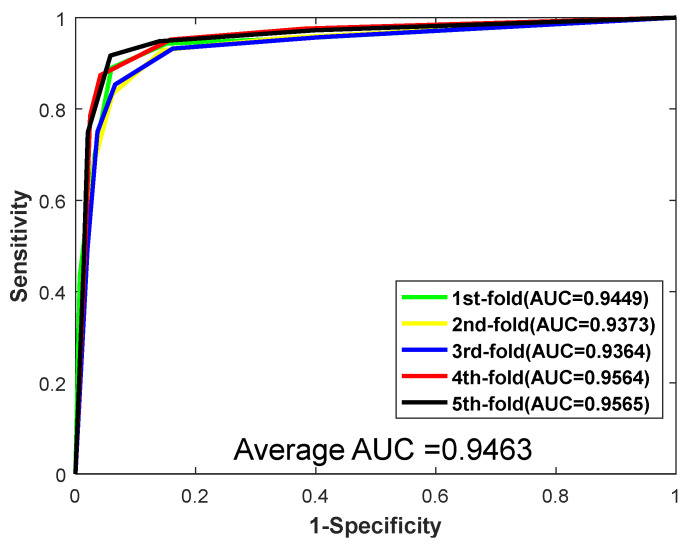
ROC curves yielded by RF on *H. pylori*.

When predicting the PPIs of the *Yeast* dataset, the proposed method yielded results of average accuracy is 92.81%, precision is 96.80%, sensitivity is 88.55%, and MCC is 86.61%, respectively. The corresponding standard deviations are 0.66%, 0.68%, 0.95%, and 1.15%, respectively. When predicting the *H. pylori*, the average accuracy, precision, sensitivity, and MCC are 92.56%, 94.11%, 90.82%, and 86.22%, with the corresponding standard deviations of 0.86%, 0.99%, 0.93%, and 1.47%, respectively. Meanwhile, the values of AUC were also computed. The ROC curves of the two datasets are shown in Figure 2 and Figure 3. As shown in Figure 2 and Figure 3, for the dataset *Yeast*, the AUC value is 0.9506. For the dataset *H. pylori*, we could clearly see that the AUC value is 0.9463. In conclusion, promising results demonstrate that the method we proposed is stable and effective for predicting PPIs.

### 3.3. Performance Comparison of RF with Other Models

**Figure 4:**
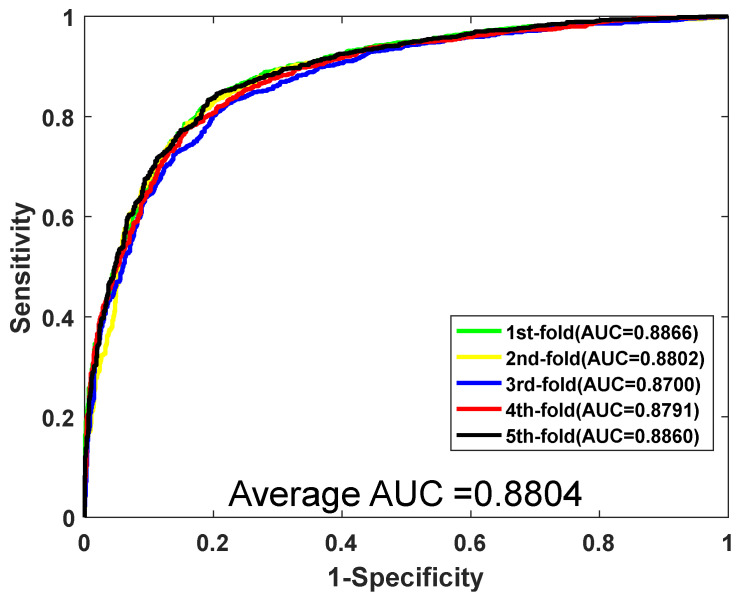
The ROC curve of the SVM classifier on the *Yeast* dataset.

**Figure 5:**
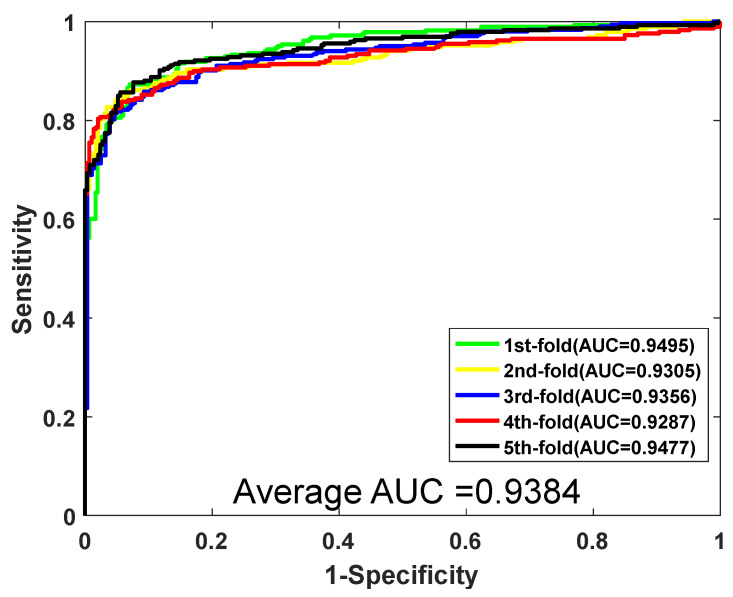
The ROC curve of the SVM classifier on the *H. pylori* dataset.

We compare the proposed method with *K* nearest neighbor (KNN) and support vector machine (SVM) classifier for further evaluating the proposed method. The algorithm of KNN is widely used in machine learning due to its efficiency and simplicity. The parameter k of KNN needs to be optimized to obtain the best performance. Here, the *k* is set to 2. When training the SVM model, the LIBSVM tool is adopted to predict PPIs. There are two corresponding parameters of *c* and *g* that need to be optimized in the SVM classifier. When we carried out the experiment using the same feature vectors, 40-dimensions in *Yeast* and 80-dimensions in *H. pylori*, in the SVM classifier, we optimized several parameters to find the best performance of the classifier. Thus, in the *Yeast* dataset, parameters *c* and *g* are set 1 and 4. In the *H. pylori* dataset, we optimize the parameter and finally, we set *c* = 5 and *g* = 0.1, respectively. The performance results of SVM can be seen in Table 4 and Table 5. When using SVM for predicting the *Yeast* PPI dataset, the performance result of average accuracy is 80.72%, precision is 81.39%, sensitivity is 79.66%, MCC is 68.87%, and AUC becomes 0.8804, respectively. The corresponding standard deviations are 0.81%, 1.16%, 0.98%, 0.98%, and 0.0067, respectively. When the SVM is adopted to predict *H. pylori*, the prediction results of average accuracy, precision, sensitivity, MCC, and AUC are 88.71%, 91.86%, 85.06%, 79.91%, and 0.9438, respectively. The ROC curves of the SVM classifier on the *Yeast* and *H. pylori* datasets are shown in Figure 4 and Figure 5.

Furthermore, the prediction experiment of KNN has been carried out and the average results are obtained by adopting the same feature extraction method. Table 6 summarizes the prediction result of the different prediction models. The results of RF are significantly better than SVM and KNN on the *Yeast* dataset. For example, the accuracy gaps between RF and SVM are 12.09% in the *Yeast* dataset and 3.85% in the *H. pylori* dataset. Similarly, the accuracy gaps between RF and KNN are 18.08% in the *Yeast* dataset and 1.51% in the *H. pylori* dataset. As a result, we can conclude that the RF classifier is more accurate and outstanding than SVM and KNN.

**Figure 6:**
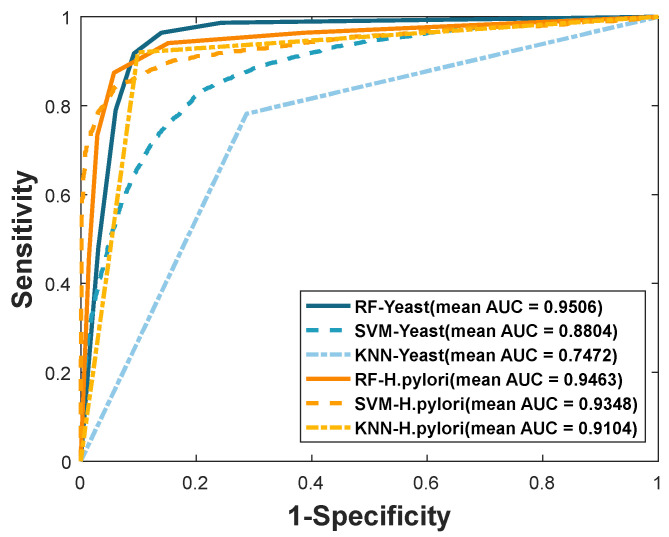
Comparison of ROC curves for different classifiers of RF, SVM, and KNN on two datasets: *Yeast* and *H. pylori*.

To evaluate the method performance more intuitively, we plotted the ROC curves of RF, SVM, and KNN, which can be seen in Figure 6. It can be known that the higher the AUC value, the better the performance of the experimental method. For instance, the AUC values gaps between RF and SVM are 0.0702 in the *Yeast* dataset and 0.0025 in the *H. pylori* dataset. Similarly, the AUC values gaps between RF and KNN are 0.2034 in the *Yeast* dataset and 0.0359 in the *H. pylori* dataset. 

Given more thinking in this section, we adopt the same feature vectors in different classifiers: KNN, SVM, and RF. SVM shows many unique advantages in solving small samples and nonlinear and high-dimensional pattern recognition. It always shows the state-of-the-art performance in many previous works. In addition, KNN is a commonly supervised learning method in which K is usually selected manually. In this study, RF obtained better accuracy results than KNN and SVM; it also demonstrates when predicting the protein–protein interactions, RF can capture more useful information and have less noise influence than SVM and KNN. Thus, we conclude that the method we proposed has better prediction performance for predicting PPIs. 

### 3.4. Performance on Independent Dataset

Although our method has achieved satisfactory results, we carried out an extensive experiment on our proposed method. Four independent PPI datasets, including *H. pylori*, *H. sapiens*, *C. elegans*, and *M. musculus*, were selected to evaluate the predictive capacity of the proposed model. The experiment is based on the hypothesis that a large number of interacting proteins in an organism evolve in a related way, and their respective orthologs in other organisms also interact. Specially, we first adopted the *Yeast* PPI dataset as the training set after optimizing the parameters. The same feature extraction method was applied to four independent PPI datasets, and then these feature vectors of the independent dataset would be treated as test data. The results are summarized in Table 7.

When applying our proposed method to predict PPIs on four cross-species, we achieved the average values of accuracy varying from 88.60% to 97.44% in Table 7. Moreover, based on our hypothesis, the accuracy result for the *H. sapiens* dataset is 88.60% and 97.44% for *M. musculus* dataset. We can suppose that when we employ the *Yeast* dataset as the training set, the *H. sapiens* dataset shows a lower correlation; on the contrary, the *M. musculus* dataset shows a higher correlation. All in all, it demonstrates that our proposed model has good predictive and generalization capabilities in predicting PPIs and can be applied to different protein interaction prediction problems.

### 3.5. Comparison with Other Methods

There have been proposed many related works for improving the prediction performance. To compare whether our method is efficient or not, we make a comparison between our method and the previous works on the *Yeast* and *H. pylori* PPI datasets. Table 8 and Table 9 list the comparison result on two datasets.

Table 8 clearly illustrates that our proposed method achieved the best results in accuracy, precision, sensitivity, and MCC, respectively. Especially in the criteria of the MCC, our method is 8.09% higher than the ensemble ELM method, and our model is 5.06% higher in accuracy than ensemble ELM. Generally, the result of our method is ranked first that our model obtained the highest prediction accuracy on the *H. pylori*.

In Table 9, the results of previous work are listed. Our approach achieved the highest result in several criteria. Specifically, the proposed method achieved 92.81% on accuracy, which is 3.48% higher than the first highest in Guo’s work. The results yielded from our model on sensitivity only achieved 88.55%, which was 9.35% lower than the third-highest in Yang’s work. It is worth noting that the feature extraction method and classifier make a great contribution to achieving excellent performance. Generally speaking, the performance of the method we proposed is superior to other methods in the table and is effective in predicting PPIs.

## 4. Conclusions

In this article, we reported a novel computational method by combining locality-preserving projections and rotation forest for inferring potential PPIs. It is worth noting that the feature extraction method is conducive to predicting PPIs. The main improvement in this work is that the locality preserving projections (LPP) are insensitive to anisotropic values and can better maintain fixed local structure information internally. Then obtaining the final prediction result by employing rotation forest. The method achieved an average prediction accuracy of 92.81% on *Yeast* and 92.56% on *H. pylori*. We also further compare the prediction performance among the rotation forest, support vector machine, and *K* nearest neighbor. Extensive experiments were also carried out on four independent datasets. The experiment results show that the performance of our model is appropriate. However, the computational method still has some drawbacks, the feature vectors extracted by LPP have potential noise, and evolutionary information cannot be retained completely. In future studies, we will continue to study the use of more efficient descriptors to predict PPIs. 

## Figures and Tables

**Table 1:** The results of different feature vectors on the *Yeast* and *H. pylori* datasets.

Feature Vectors	Dataset	Acc. (%)	Prec. (%)	Sen. (%)	MCC. (%)
40	*Yeast*	**92.81 ± 0.66**	**96.80 ± 0.68**	**88.55 ± 0.95**	**86.61 ± 1.15**
	*H. pylori*	92.18 ± 0.70	93.66 ± 2.21	90.56 ± 1.52	85.54 ± 1.15
60	*Yeast*	92.55 ± 0.32	96.56 ± 0.53	88.25 ± 0.81	86.16 ± 0.31
	*H. pylori*	92.49 ± 2.18	94.59 ± 2.13	90.12 ± 2.59	86.16 ± 3.67
80	*Yeast*	92.60 ± 0.32	96.37 ± 0.55	88.51 ± 0.54	86.23 ± 0.57
	*H. pylori*	**92.56 ± 0.86**	**94.11 ± 0.99**	**90.82 ± 0.93**	**86.22 ± 1.47**
100	*Yeast*	91.90 ± 0.44	94.94 ± 0.90	88.52 ± 0.46	85.08 ± 0.73
	*H. pylori*	92.21 ± 1.19	94.10 ± 1.74	90.12 ± 2.31	85.63 ± 2.03
120	*Yeast*	92.56 ± 0.75	96.44 ± 0.79	88.40 ± 0.93	86.19 ± 1.27
	*H. pylori*	91.90 ± 1.66	93.94 ± 1.14	89.56 ± 2.56	85.14 ± 2.81
140	*Yeast*	92.52 ± 0.48	95.96 ± 0.32	88.77 ± 0.79	86.12 ± 0.81
	*H. pylori*	91.46 ± 1.09	92.74 ± 2.54	89.89 ± 1.84	84.34 ± 1.84

**Table 2:** Prediction performance of the *Yeast* dataset based on five-fold cross-validation method.

Testing Set	Acc. (%)	Prec. (%)	Sen. (%)	MCC. (%)	AUC
1	92.58	97.34	88.14	86.23	0.9509
2	92.80	96.24	88.78	86.58	0.9502
3	92.85	97.39	88.35	86.68	0.9511
4	92.00	95.91	87.44	85.20	0.9472
5	93.83	97.13	90.02	88.37	0.9535
**Average**	**92.81 ± 0.66**	**96.80 ± 0.68**	**88.55 ± 0.95**	**86.61 ± 1.15**	**0.9506 ± 0.0023**

**Table 3:** Prediction performance of the *H. pylori* dataset based on five-fold cross-validation method.

Testing Set	Acc. (%)	Prec. (%)	Sen. (%)	MCC. (%)	AUC
1	92.80	93.50	91.52	86.61	0.9449
2	91.77	93.19	89.97	84.87	0.9373
3	91.60	93.49	90.10	84.59	0.9364
4	93.65	95.02	92.07	88.10	0.9564
5	92.97	95.32	90.44	86.91	0.9565
**Average**	**92.56 ± 0.86**	**94.11 ± 0.99**	**90.82 ± 0.93**	**86.22 ± 1.47**	**0.9463 ± 0.0098**

**Table 4:** Prediction performance of the *Yeast* dataset based on five-fold cross-validation method.

Testing Set	Acc. (%)	Prec. (%)	Sen. (%)	MCC. (%)	AUC
1	81.27	83.41	79.90	69.53	0.8866
2	81.18	81.28	80.02	69.42	0.8802
3	79.48	80.85	78.37	67.37	0.8700
4	80.33	80.54	79.07	68.37	0.8791
5	81.36	80.88	80.95	69.65	0.8860
**Average**	**80.72 ± 0.81**	**81.39 ± 1.16**	**79.66 ± 0.98**	**68.87 ± 0.98**	**0.8804 ± 0.0067**

**Table 5:** Prediction performance of *H. pylori* dataset based on five-fold cross-validation method.

Testing Set	Acc. (%)	Prec. (%)	Sen. (%)	MCC. (%)	AUC
1	88.16	88.21	87.28	79.11	0.9495
2	89.37	92.83	85.12	80.91	0.9305
3	87.65	90.81	84.82	78.32	0.9356
4	88.85	94.47	82.41	80.02	0.9287
5	89.54	92.96	85.67	81.21	0.9477
**Average**	**88.71 ± 0.80**	**91.86 ± 2.42**	**85.06 ± 1.76**	**79.91 ± 1.21**	**0.9384 ± 0.0097**

**Table 6:** The experimental results compared with other prediction models in the *Yeast* and *H. pylori* datasets.

Dataset	Model	Accu. (%)	Prec. (%)	Sen. (%)	MCC. (%)	AUC
*Yeast*	**RF**	**92.81 ± 0.66**	**96.80 ± 0.68**	**88.55 ± 0.95**	**86.61 ± 1.15**	**0.9506 ± 0.0023**
	SVM	80.72 ± 0.81	81.39 ± 1.16	79.66 ± 0.98	68.87 ± 0.98	0.8804 ± 0.0067
	KNN	74.73 ± 1.38	76.57 ± 2.18	71.28 ± 1.18	62.15 ± 1.31	0.7472 ± 0.0139
*H. pylori*	**RF**	**92.56 ± 0.86**	**94.11 ± 0.99**	**90.82 ± 0.93**	**86.22 ± 1.47**	**0.9463 ± 0.0098**
	SVM	88.71 ± 0.80	91.86 ± 2.42	85.06 ± 1.76	79.91 ± 1.21	0.9384 ± 0.0097
	KNN	91.05 ± 1.01	91.85 ± 1.72	90.12 ± 0.94	83.70 ± 1.64	0.9104 ± 0.0101

**Table 7:** Prediction results were obtained on four independent datasets.

Species	Test Pairs	Accu. (%)
*H. sapiens*	1412	**88.60**
*M. musculus*	313	**97.44**
*H. pylori*	1420	**94.44**
*C. elegans*	4013	**93.60**

**Table 8:** Comparison results of different methods on *H. pylori*.

Model	Acc. (%)	Prec. (%)	Sen. (%)	MCC. (%)
Ensemble of HKNN [47]	86.60	85.00	86.70	N/A
HKNN [48]	84.00	84.00	86.00	N/A
Ensemble ELM [49]	87.50	88.95	86.15	78.13
Signature products [40]	83.40	85.70	79.90	N/A
Phylogenetic bootstrap [50]	75.80	80.20	69.80	N/A
Boosting [51]	79.52	81.69	80.37	70.64
**Proposed method**	**92.56**	**94.11**	**90.82**	**86.22**

**Table 9:** Comparison results of different methods on *Yeast*.

Method	Model	Acc. (%)	Prec. (%)	Sen. (%)	MCC. (%)
You’s work [49]	PCA-EELM	87.00 ± 0.29	87.59 ± 0.32	86.15 ± 0.43	77.36 ± 0.44
Zhou’s work [52]	SVM+LD	88.56 ± 0.33	89.50 ± 0.60	87.37 ± 0.22	77.15 ± 0.68
Yang’s work [53]	Cod1	75.08 ± 1.13	74.75 ± 1.23	75.81 ± 1.20	N/A
	Cod2	80.04 ± 1.06	95.44 ± 0.30	96.25 ± 1.26	N/A
	Cod3	80.41 ± 0.47	65.50 ± 1.44	97.90 ± 1.06	N/A
	Cod4	86.15 ± 1.17	90.24 ± 1.34	81.03 ± 1.74	N/A
Guo’s work [54]	ACC	89.33 ± 2.67	88.87 ± 6.16	89.93 ± 3.68	N/A
	AC	87.36 ± 1.38	87.82 ± 4.33	87.30 ± 4.68	N/A
**Proposed method**	**RF**	**92.81 ± 0.66**	**96.80 ± 0.68**	**88.55 ± 0.95**	**86.61 ± 1.15**

## Data Availability

All the data are available at https://github.com/TorchZhan/LPP_PPI (accessed on 29 June 2022).

## References

[1] Wang L., You Z.H., Chen X., Li J.Q., Yan X., Zhang W., Huang Y.A. (2017). An ensemble approach for large-scale identification of protein-protein interactions using the alignments of multiple sequences. Oncotarget.

[2] Braun P., Gingras A.C. (2012). History of protein-protein interactions: From egg-white to complex networks. Proteomics.

[3] Takashi I., Tomoko C., Ritsuko O., Mikio Y., Masahira H., Yoshiyuki S. (2001). A comprehensive two-hybrid analysis to explore the yeast protein interactome. Proc. Natl. Acad. Sci. USA.

[4] Tarassov K., Messier V., Landry C.R., Radinovic S., Molina M.M.S., Shames I., Malitskaya Y., Vogel J., Bussey H., Michnick S.W. (2008). An in Vivo Map of the Yeast Protein Interactome. Science.

[5] Zhu H., Snyder M. (2003). Protein chip technology. Curr. Opin. Chem. Biol..

[6] Gavin A.C., Bosche M., Krause R., Grandi P., Marzioch M., Bauer A., Schultz J., Rick J.M., Michon A.M., Cruciat C.M. (2002). Functional organization of the yeast proteome by systematic analysis of protein complexes. Nature.

[7] Bader G.D., Doron B., Hogue C.W. (2003). BIND: The biomolecular interaction network database. Nucleic Acids Res..

[8] Xenarios I., Salwinski L., Duan X.J., Higney P., Kim S.M., Eisenberg D. (2002). DIP, the Database of Interacting Proteins: A research tool for studying cellular networks of protein interactions. Nucleic Acids Res..

[9] Licata L., Briganti L., Peluso D., Perfetto L., Iannuccelli M., Galeota E., Sacco F., Palma A., Nardozza A.P., Santonico E. (2012). MINT, the molecular interaction database: 2012 update. Nucleic Acids Res..

[10] Zhu L., You Z.H., Huang D.S., Wang B. (2013). T-LSE: A Novel Robust Geometric Approach for Modeling Protein-Protein Interaction Networks. PLoS ONE.

[11] Cui G.Y.C., Chen Y., Huang D.S., Han K. (2008). An algorithm for finding functional modules and protein complexes in protein-protein interaction networks. J. Biomed. Biotechnol..

[12] Xia J.F., Zhao X.M., Huang D.S. (2010). Predicting protein-protein interactions from protein sequences using meta predictor. Amino Acids.

[13] Li J.J., Huang D.S., Wang B., Chen P. (2006). Identifying Protein-Protein Interfacial Residues in Heterocomplexes Using Residue Conservation Scores. Int. J. Biol. Macromol..

[14] Shi M.G., Xia J.F., Li X.L., Huang D.S. (2010). Predicting protein-protein interactions from sequence using correlation coefficient and high-quality interaction dataset. Amino Acids.

[15] Chen P., Wang B., Wong H.S., Huang D.S. (2007). Prediction of protein B-factors using multi-class bounded SVM. Protein Pept. Lett..

[16] Zhao X.M., Cheung Y.M., Huang D.S. (2005). A novel approach to extracting features from motif content and protein composition for protein sequence classification. Neural Netw..

[17] Zhu L., Deng S.P., You Z.H., Huang D.S. (2017). Identifying spurious interactions in the protein-protein interaction networks using local similarity preserving embedding. IEEE/ACM Trans. Comput. Biol. Bioinform..

[18] Bao W.Z., Yuan C.A., Zhang Y.H., Han K., Nandi A.K., Honig B., Huang D.S. (2018). Mutli-features prediction of protein translational modification sites. IEEE/ACM Trans. Comput. Biol. Bioinform..

[19] Xia J.F., Zhao X.M., Song J.N., Huang D.S. (2010). APIS: Accurate prediction of hot spots in protein interfaces by combining protrusion index with solvent accessibility. BMC Bioinform..

[20] Wang Y.B., You Z.H., Li L.P., Huang D.S., Zhou F.F., Yang S. (2018). Improving prediction of self-interacting proteins using stacked sparse auto-encoder with PSSM profiles. Int. J. Biol. Sci..

[21] Deng S.P., Huang D.S. (2014). SFAPS: An R package for structure/function analysis of protein sequences based on informational spectrum method. Methods.

[22] Huang D.S., Zhang L., Han K., Deng S.P., Yang K., Zhang H.B. (2014). Prediction of protein-protein interactions based on protein-protein correlation using least squares regression. Curr. Protein Pept. Sci..

[23] Wang B., Huang D.S., Jiang C.J. (2014). A new strategy for protein interface identification using manifold learning method. IEEE Trans. Nano-Biosci..

[24] Lei Y.K., You Z.H., Ji Z., Zhu L., Huang D.S. (2012). Assessing and predicting protein interactions by combining manifold embedding with multiple information integration. BMC Bioinform..

[25] You Z.H., Lei Y.K., Huang D.S., Zhou X.B. (2010). Using manifold embedding for assessing and predicting protein interactions from high-throughput experimental data. Bioinformatics.

[26] Alguwaizani S., Park B., Zhou X., Huang D.S., Han K. (2018). Predicting interactions between virus and host proteins using repeat patterns and composition of amino acids. J. Healthc. Eng..

[27] Yi H.C., You Z.H., Huang D.S., Li X., Jiang T.H., Li L.P. (2018). A deep learning framework for robust and accurate prediction of ncRNA-protein interactions using evolutionary information. Mol. Ther. Nucleic Acids.

[28] Huang D.S., Zhao X.M., Huang G.B., Cheung Y.M. (2006). Classifying protein sequences using hydropathy blocks. Pattern Recognit..

[29] Wang B., Chen P., Huang D.S., Li J.J., Lok T.M., Lyu M.R. (2006). Predicting protein interaction sites from residue spatial sequence profile and evolution rate. FEBS Lett..

[30] Zhao X.M., Huang D.S., Cheung Y.M. (2005). A novel hybrid GA/RBFNN technique for protein classification. Protein Pept. Lett..

[31] Wang B., Wong H.S., Huang D.S. (2006). Inferring protein-protein interacting sites using residue conservation and evolutionary information. Protein Pept. Lett..

[32] Xia J.F., Han K., Huang D.S. (2010). Sequence-based prediction of protein-protein interactions by means of rotation forest and autocorrelation descriptor. Protein Pept. Lett..

[33] Shen J., Zhang J., Luo X., Zhu W., Yu K., Chen K., Li Y., Jiang H. (2007). Predicting protein–protein interactions based only on sequences information. Proc. Natl. Acad. Sci. USA.

[34] You Z.H., Zhu L., Zheng C.H., Yu H.J., Deng S.P., Ji Z. (2014). Prediction of protein-protein interactions from amino acid sequences using a novel multi-scale continuous and discontinuous feature set. BMC Bioinform..

[35] Chen C., Zhang Q., Yu B., Yu Z., Lawrence P.J., Ma Q., Zhang Y. (2020). Improving protein-protein interactions prediction accuracy using XGBoost feature selection and stacked ensemble classifier. Comput. Biol. Med..

[36] Zhao L.J., Yuan D.C., Chai T.Y., Tang J. (2011). KPCA and ELM ensemble modeling of wastewater effluent quality indices. Procedia Eng..

[37] Yousef A., Charkari N.M. (2013). A novel method based on new adaptive LVQ neural network for predicting protein–protein interactions from protein sequences. J. Theor. Biol..

[38] Wang T., Li L., Huang Y.A., Zhang H., Ma Y., Zhou X. (2018). Prediction of protein-protein interactions from amino acid sequences based on continuous and discrete wavelet transform features. Molecules.

[39] Zahiri J., Yaghoubi O., Mohammad-Noori M., Ebrahimpour R., Masoudi-Nejad A. (2013). PPIevo: Protein–protein interaction prediction from PSSM based evolutionary information. Genomics.

[40] Martin S., Roe D., Faulon J.L. (2005). Predicting protein–protein interactions using signature products. Bioinformatics.

[41] Gribskov M., McLachlan A.D., Eisenberg D. (1987). Profile analysis: Detection of distantly related proteins. Proc. Natl. Acad. Sci. USA.

[42] Huang C., Yuan J. (2013). Using radial basis function on the general form of Chou’s pseudo amino acid composition and PSSM to predict subcellular locations of proteins with both single and multiple sites. Biosystems.

[43] Verma R., Varshney G.C., Raghava G.P.S. (2010). Prediction of mitochondrial proteins of malaria parasite using split amino acid composition and PSSM profile. Amino Acids.

[44] Altschul S.F., Madden T.L., Schaffer A.A., Zhang J., Zhang Z., Miller W., Lipman D.J. (1997). Gapped BLAST and PSI-BLAST: A new generation of protein database search programs. Nucleic Acids Res..

[45] He X., Niyogi P. (2004). Locality preserving projections. Adv. Neural Inf. Process. Syst..

[46] Rodriguez J.J., Kuncheva L.I., Alonso C.J. (2006). Rotation forest: A new classifier ensemble method. IEEE Trans. Pattern Anal. Mach. Intell..

[47] Nanni L., Lumini A. (2006). An ensemble of K-local hyperplanes for predicting protein–protein interactions. Bioinformatics.

[48] Nanni L. (2005). Hyperplanes for predicting protein–protein interactions. Neurocomputing.

[49] You Z.H., Lei Y.K., Zhu L., Xia B., Wang B. (2013). Prediction of protein-protein interactions from amino acid sequences with ensemble extreme learning machines and principal component analysis. BMC Bioinform..

[50] Bock J.R., Gough D.A. (2003). Whole-proteome interaction mining. Bioinformatics.

[51] Liu B., Yi J., Aishwarya S.V., Lan Y., Ma Y., Huang T.H., Leone G., Jin V.X. (2013). QChIPat: A quantitative method to identify distinct binding patterns for two biological ChIP-seq samples in different experimental conditions. BMC Genom..

[52] Zhou Y.Z., Gao Y., Zheng Y.Y. (2011). Prediction of protein-protein interactions using local description of amino acid sequence. Adv. Comput. Sci. Educ. Appl..

[53] Yang L., Xia J.F., Gui J. (2010). Prediction of protein-protein interactions from protein sequence using local descriptors. Protein Pept. Lett..

[54] Guo Y.Z., Yu L.Z., Wen Z.N., Li M.L. (2008). Using support vector machine combined with auto covariance to predict protein-protein interactions from protein sequences. Nucleic Acids Res..

